# Cultural Modulation of Face and Gaze Scanning in Young Children

**DOI:** 10.1371/journal.pone.0074017

**Published:** 2013-08-26

**Authors:** Atsushi Senju, Angélina Vernetti, Yukiko Kikuchi, Hironori Akechi, Toshikazu Hasegawa

**Affiliations:** 1 Centre for Brain and Cognitive Development, Birkbeck, University of London, London, United Kingdom; 2 Japan Society for the Promotion of Science, Tokyo, Japan; 3 College of Education, Ibaraki University, Ibaraki, Japan; 4 Division of Information System Design, Tokyo Denki University, Saitama, Japan; 5 Department of Cognitive and Behavioral Science, University of Tokyo, Tokyo, Japan; Ecole Normale Supérieure, France

## Abstract

Previous research has demonstrated that the way human adults look at others’ faces is modulated by their cultural background, but very little is known about how such a culture-specific pattern of face gaze develops. The current study investigated the role of cultural background on the development of face scanning in young children between the ages of 1 and 7 years, and its modulation by the eye gaze direction of the face. British and Japanese participants’ eye movements were recorded while they observed faces moving their eyes towards or away from the participants. British children fixated more on the mouth whereas Japanese children fixated more on the eyes, replicating the results with adult participants. No cultural differences were observed in the differential responses to direct and averted gaze. The results suggest that different patterns of face scanning exist between different cultures from the first years of life, but differential scanning of direct and averted gaze associated with different cultural norms develop later in life.

## Introduction

Faces are often described as a ‘window to the soul’, because facial expressions signal a person’s emotional state, and gaze direction can tell you what they see and what they know. Attention to facial features can be observed in the first few hours or days of life. Newborns preferentially orient to faces [1,2], especially those with direct gaze [3]. Eye-tracking studies have demonstrated that infants start to show adult-like face scanning behaviour, such as preferential fixations on the eyes and mouth [4], from as early as 6 weeks after birth [5]. Atypical patterns of face scanning behaviour can be found in neurodevelopmental disorders, such as autism spectrum disorders (ASD), whereby individuals show profound difficulties in social interaction and communication [6]. Although the mechanisms underlying atypical face scanning behaviour in ASD are still unclear, it highlights the potential relationship between face scanning behaviour and the development of social skills.

An important question concerning the development of face gaze is the role of the postnatal environment. Several major theories of the development of social skills emphasize the role of input from parents/caregivers, as well as input from other members of society; contributions which are essential for the infant brain to learn about the social world and become an ‘expert’ [7,8]. However, relatively little is known about the effect of postnatal environment on the development of face gaze, because is impossible to totally control the exposure to others in human studies. To overcome this challenge, several lines of research have utilized eye-tracking techniques to contrast face gaze between participants from Western European / North American culture and Eastern Asian cultures, testing how different cultural norms might systematically affect the development of face gaze.

Firstly, one series of studies recorded the eye movements of Western European (British) and Eastern Asian (mainly Chinese) adults as they processed static image of faces, and found that Western European participants showed triangular fixation, taking in both the eyes and the mouth, but Eastern Asian participants fixated more on the centre of the face [9,10]. It was also suggested that reduced fixation on the mouth could partly explain cross-cultural difference in facial expression processing [11]. Recently, Kelly et al. [12] replicated the same culture-specific pattern of fixation in children aged 7-12 years. In addition, two studies published from the same group reported the face gaze behaviour of Caucasian [13] and Eastern Asian [14] infants in their first year of life. Data from these studies suggested that a similar trend could be present even in young infants, but direct cross-cultural comparison between these two populations were not reported. These studies clearly show the subtle differences in face fixations between the participants with different cultural backgrounds, which could develop from early in infancy. However, it is not clear whether these effects of culture are specific to the context they analyse facial information from; static or dynamic images with little meaningful facial actions, or more general patterns of fixations in more realistic contexts where they see dynamic sequences of facial actions such as facial expression and gaze shift.

Secondly, Senju et al. [15] recorded the eye movements of Western European (British) and Eastern Asian (Japanese) adults while they observed dynamic faces presenting different facial actions, such as changing gaze direction (looking towards or away from the observer) and changing mouth movement (smiling or opening mouth). They reported more fixations on the mouth area in British than in Japanese participants, and more fixations on the eyes in Japanese than in British participants, replicating other studies using static images of faces [9–11]. However, they did not replicate the finding of Eastern Asian participants having longer fixations on the central parts of faces [9,10]. They also reported differential effects of stimuli gaze direction upon face gaze behaviour across cultures; British participants were less affected by the gaze shift than Japanese participants, who shifted their fixation to the corresponding gaze direction of the stimulus face. These results are generally consistent with McCarthy et al. [16,17], who reported that Caucasian adults tended to hold eye contact with an interviewer for longer durations when answering cognitively demanding questions, while Eastern Asian (Japanese) adults broke eye contact relatively easily in the same situation (but see also 18). Conversely, changes in mouth movements did not affect face gaze in either population in Senju et al. [15]. Furthermore, this effect found in Senju et al. [15] was more pronounced in males than in females, suggesting that the effects of cultural norms on face and gaze processing are manifested more strongly in males. Further studies are required to see how differences in gender-related cultural norm interact with face-scanning behaviour. However, no studies have been conducted to study how such culture-specific pattern of the face gaze develops over the course of time.

The current study aimed to test the culture-specific pattern of face gaze in response to a dynamic display containing multiple face actions, using similar paradigm as in Senju [15]. The age range of 1-7 years was selected because it is the approximate age at which a child starts attending nursery and/or kindergarten, and thus starts encountering peers and other adults more frequently. Two different predictions can be made about the effects of culture on the developmental course of face gaze. Firstly, if the cultural effects in the face gaze develop in the first years of life [13,14], we should replicate the adults’ results in even the youngest participants. Secondly, if the effects of cultural norms develop much later, we should see similar face gaze in children in both cultures. We focused our analyses on (1) the differential fixation to eyes and mouth, and (2) differential response to different gaze direction, based on previous finding in the adults.

## Methods

**Figure 1:**
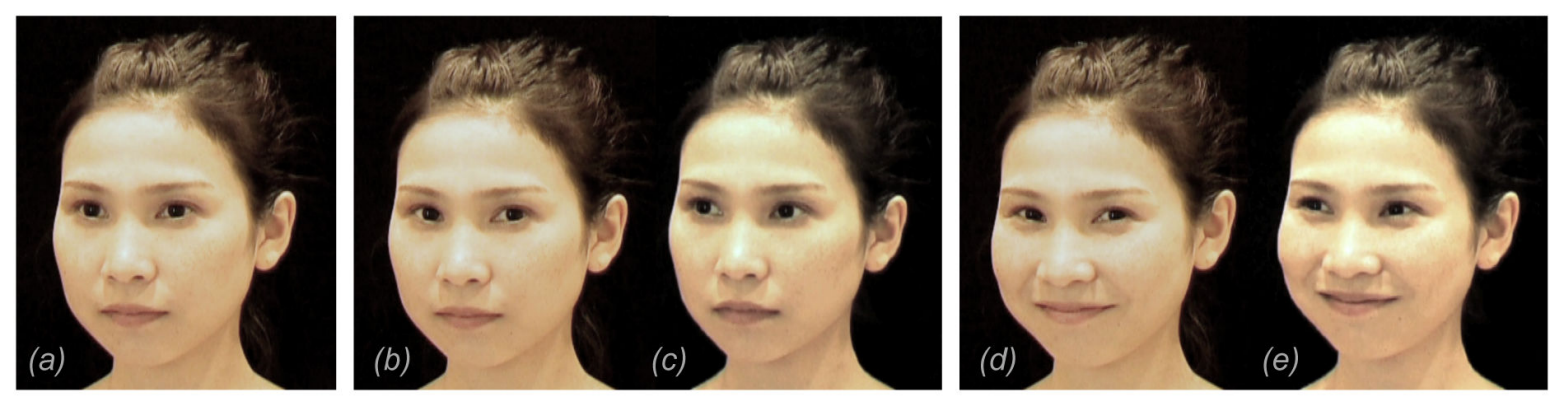
Sample of the gaze direction of the stimuli. All the actors initially showed (a) forward gaze and closed mouth. After one second, the actor shifted her gaze (b) toward the observer or (c) away from him/her. After another one second (i.e., 2 seconds from the onset of the stimulus), the face smiled (d and e). After that, the face remained still for another three seconds. The orientation of the face was right in half of the stimuli and the left in the other half.

**Figure 2:**
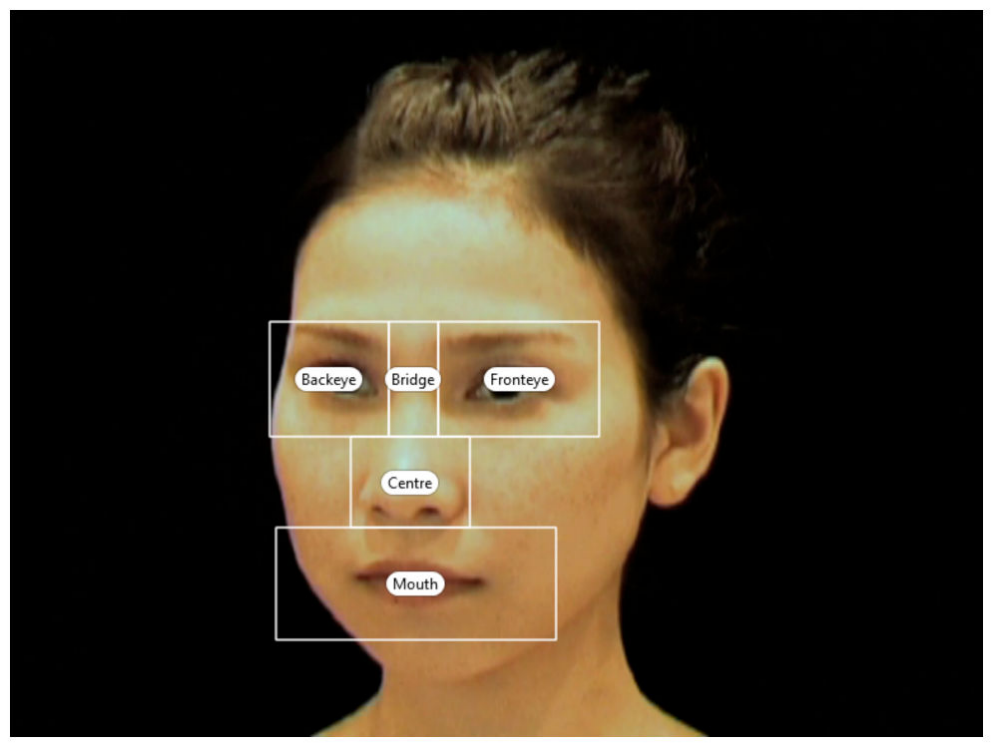
Examples of the area of interest (AOI); front eye, back eye, bridge, centre and mouth. The size and the location of the AOI were constant across different stimuli.

36 British children of Caucasian ethnic background (19 females and 17 males, mean age 4.03 years, standard deviation (SD) = 1.70, range: 1-7 years) and 36 Japanese children with Japanese ethnic background (19 females and 17 males, mean age 4.08 years, standard deviation (SD) = 1.60, range: 1-7 years) participated in the study (5 children were excluded because of excessive eye tracker data loss, samples under 50%). British children were recruited in central London, and Japanese children were recruited in central Tokyo. All the participants had normal or corrected-to-normal acuity. The experiments in this study were conducted according to the Declaration of Helsinki and the procedure was approved by the Departmental Research Ethics Committee of the Department of Psychological Science, Birkbeck, University of London and by Department of Cognitive and Behavioral Sciences, University of Tokyo. Written informed consent has been obtained from the parents of all the participants. We also obtained the written consent for the use of Figures 1 and 2 under the Creative Commons license.

Four female actors (two Caucasian and two Eastern Asian) were filmed to create 6-second stimuli. All these sequences of actors’ facial actions started with a face presented upright, turned 30^°^ to the left or to the right, and gazing forward, followed by an eye movement (1 second after the start) and a smile (2 seconds after the start). Eye movements were either direct gaze (Figure 1a) or averted gaze (Figure 1b), which involved rotating both eyes laterally by 29^°^ either towards (direct gaze) or away from (averted gaze) the centre. The amount of rotation for direct gaze was selected based on the rating of 10 naïve observers, who rated the perception of ‘directedness’ of the gaze [19]. The same amount of rotation to the opposite direction was used for averted gaze. After filming, all the sequences were edited with Final Cut Express 3.0 (Apple Inc., US) to control for the overall luminance and the size of the face on the screen, as well as the exact timing of the onset of eye and mouth movements. In total, 16 sequences were created (4 faces, 2 gaze directions and 2 face orientations). The faces extended 21.5 x 18.0 cm on the screen.

Two Tobii eye-trackers (Tobii, Stockholm, Sweden) were used to present stimuli and record eye-movement in London and in Tokyo. Tobii TX 300 was used in London, with Tobii Studio software used to control stimulus presentation and to analyse the gaze data. Tobii 2150 was used in Tokyo, with Clearview software used for stimulus control and to analyse the gaze data.

Recordings were conducted in quiet rooms at each research site. Participants were instructed to watch the sequences. The same experimenter (AV) conducted the recording in both the UK and Japan, to maintain strictly similar experimental conditions such as instructions. A 5-point calibration was conducted before the recording. Recording consisted of up to four blocks, and each of 16 animations was presented once in each block, in a pseudo-randomized order. Only those children who completed 2 or more blocks of trials were included in the analyses. An experimenter joined the participant in the testing room, and was sat out of sight. Viewing distance was approximately 60-65 cm from the display.

The gaze data were initially processed with Tobii software to calculate the total visit duration (Tobii Studio for the British data and Clearview for the Japanese data). Each software applied the same filter (10 pixels fixation radius) to the raw data. Then, we calculated the visit duration for each stimulus, using the following areas of interest (AOIs); front eye, back eye, bridge, centre and mouth, as in Senju et al. [15]. Note that faces are tilted either to the right or to the left, one of the eyes is always closer to the observer (i.e. Front Eye) than the other eye (i.e. Back Eye), the latter which is off to the side (Figure 2). This visit duration data was extracted for statistical analyses.

The gaze data for different head orientations and different blocks were averaged together. To further control for the difference between different faces, the visit durations for each AOI were then divided by the total visit duration of the whole face, to calculate the relative visit duration. The relative visit duration was analysed with ANOVAs to test the effects of cultural background (British or Japanese), of sex (male or female) and of participants’ age (1-7 years), as well as the ethnicity (Caucasian or Eastern Asian), gaze direction (direct or averted) and the AOI (front eye, back eye, bridge, centre and mouth) of the stimuli. An initial ANOVA was conducted using the whole 6-second data sequence, which was then followed up with analyses of the 6 individual 1-second bins of data. To ensure robust data, post-hoc analyses (Wilcoxon sign-rank tests, and Bonferroni correction for multiple testing) were employed for each contrast.

## Results

**Figure 3:**
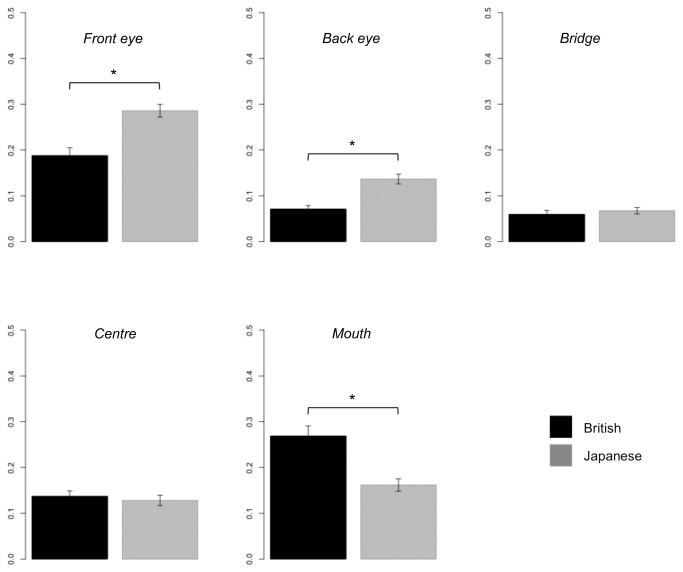
Relative visit duration on each AOI during the entire period of stimulus presentation, for each cultural background. Note. * p < .05 (corrected); error bar: standard error.

In analyses of the complete 6-second segments, there was a significant interaction between the AOI and the participants’ cultural background (*F*(4,284) = 8.629, *p* < .001, *η* ^2^_*p*_ = 0.09), replicating the adults’ data reported in Senju et al. [15]. To examine whether British participants fixate more on the mouth while Japanese participants fixate more on the eyes, as in the adult-data, we compared the relative visit duration in each AOI across participants’ cultural background. The children’s data replicated the adults’ findings; with the relative visit duration on the front eye and the back eye being significantly longer in Japanese children than in British children, and the relative visit duration on the mouth being significantly longer in British children than in Japanese children (Figure 3, all *p* < .05 (corrected)).

Unlike the adults, the present results showed no significant interactions with the actors’ gaze direction (all *F* < 1.1, all *p* > 0.35), suggesting that the gaze direction of the actor does not affect the face gaze behaviour of the children, either in British or Japanese populations.

The analyses also found a significant interaction between the AOI and the participants’ cultural background and sex (*F*(4,284) = 11.992, *p* < .001, *η* ^2^_*p*_ = 0.01), again replicating the adults’ data reported in Senju et al. [15]. For the present population, the effect was further modulated by participants’ age (*F*(4,284) = 34.314, *p* < .001, *η* ^2^_*p*_ = 0.05), suggesting the developmental change of culture-specific pattern of face gaze.

**Figure 4:**
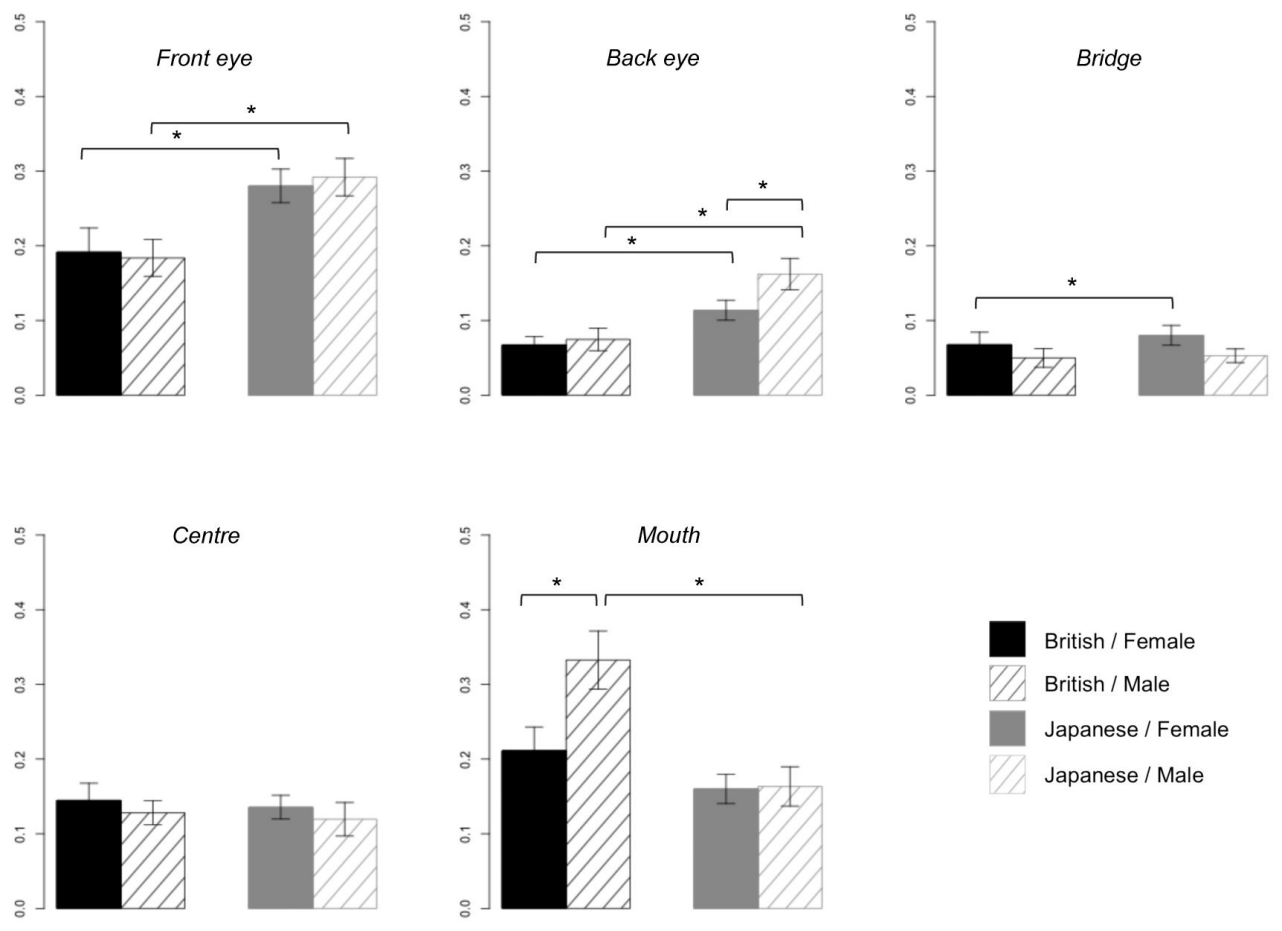
Relative visit duration on each AOI during the entire period of stimulus presentation, for each cultural background and gender of the participants. Note. * p < .05 (corrected); error bar: standard error.

To examine whether the culture-specific pattern of face gaze was more prominent in males than in females as in adult-study [15], we compared the relative visit duration in each AOI across participants’ cultural background and sex. Unlike the adults, sex of the participants did not influence data/interact with culture, with both male and female children showing significant differences between cultures (Figure 4, all *p* < .05 (corrected)). In contrast, findings of relative visit duration to the mouth fully replicated adults’ results; with only male participants showing the longer visit times to the mouth typified by participants from a British, not Japanese, cultural background (Figure 4)

**Figure 5:**
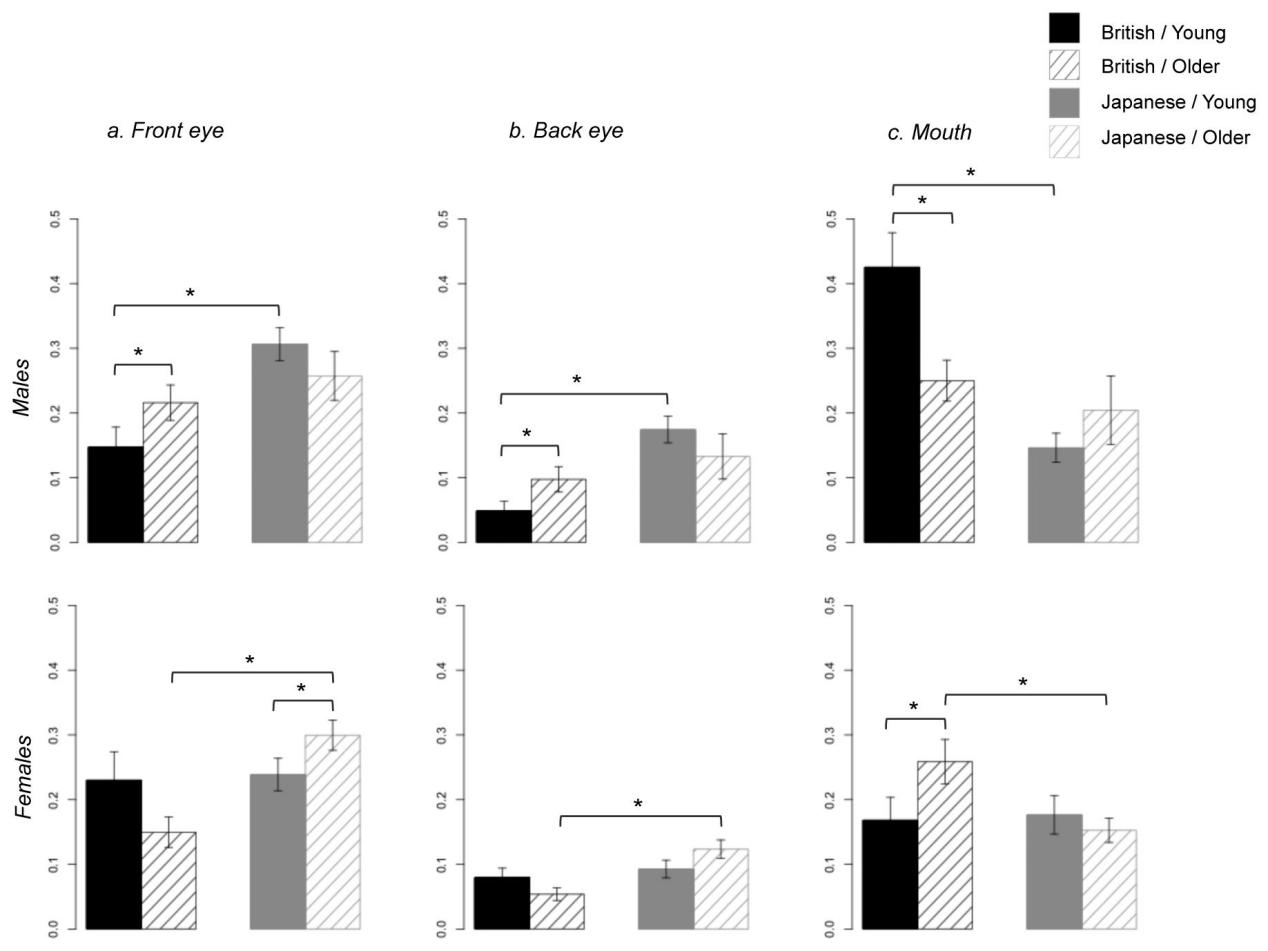
Relative visit duration between young and older children on the AOIs in (a) front eye, (b) back eye and (c) mouth during the entire period of stimulus presentation, for each cultural background and gender of the participants. Note. * p < .05 (corrected); error bar: standard error.

We then analysed developmental changes in culture-specific face gaze by contrasting younger and older participants (split median) on their gaze times for the three AOIs where we found effects of cultural background (front eye, back eye and mouth) for each of participants’ cultural background and the sex (Figure 5a, 5b, 5c). The results revealed that the culture-specific differences in face gaze were most exaggerated in younger male participants, and somewhat unexpectedly, older female participants. Such age- and gender-related changes in face gaze tended to be more prominent in British children, especially in male participants. (, all *p* < .05 (corrected)).

**Figure 6:**
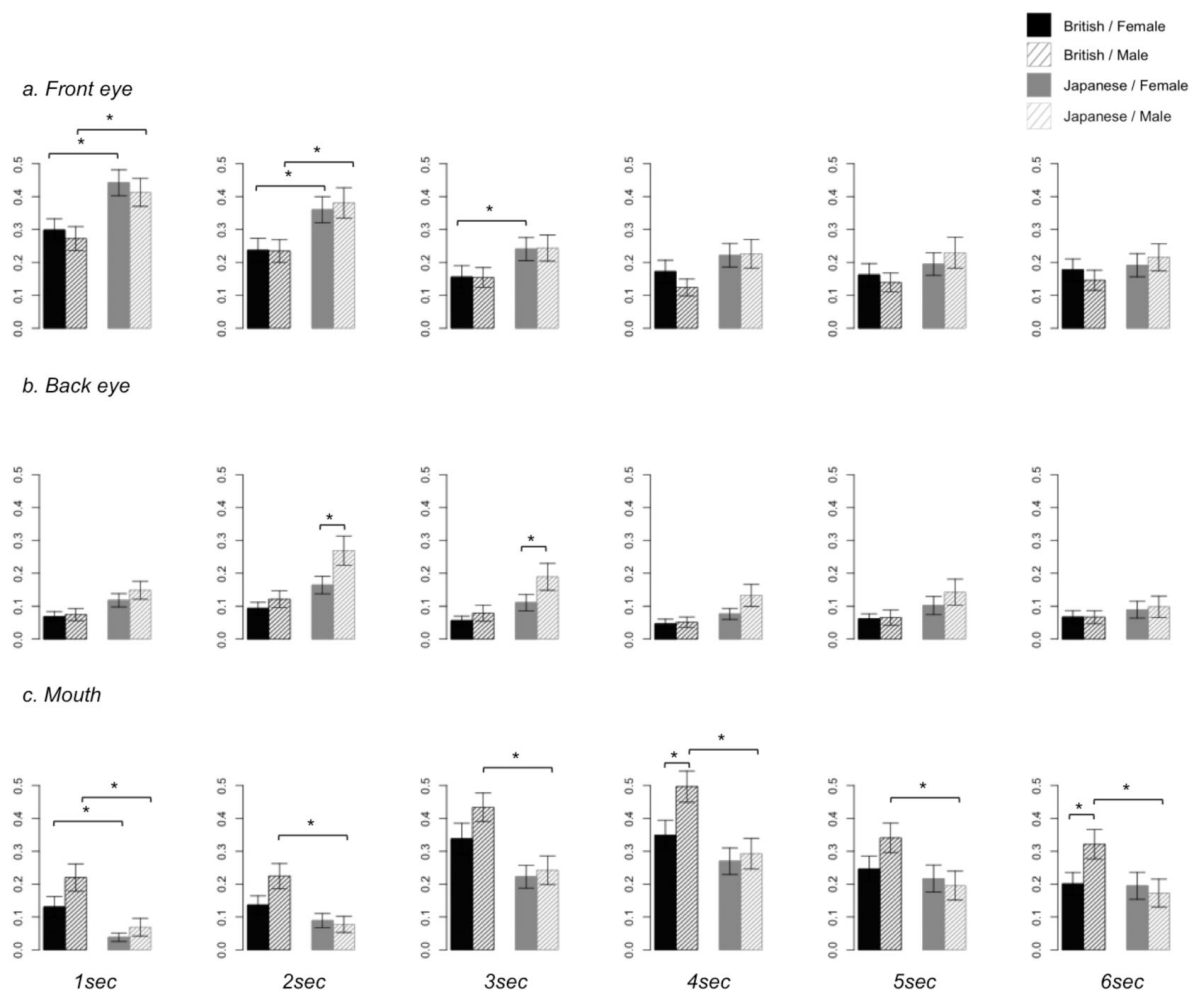
Relative visit duration on the AOIs considered (front eye, back eye and mouth) for each 1-second bin of stimulus presentation, for each cultural background and sex of the participants. Note. (a) front eye, (b) back eye and (c) mouth. * p < .05 (corrected); error bar: standard error.

Further analyses were conducted on the six 1-second bins of data (Figure 6), to explore the time-course of the differential relative visit durations on the three AOIs showing sex and cultural background interactions; front eye, back eye and mouth.

### Front eye

The effects of cultural background were significant in both male and female participants in the first and second bins, with Japanese children showing longer relative visit durations. The effect remained significant only in females in the third bin. No group effects remained significant after the fourth bin.

### Back eye

There were significant differences between male and female participants in the Japanese population, with females showing longer relative visit durations to the back eye. In the second and third bins, there were marginal effects of cultural background in male participants, which did not reach significance.

### Mouth

In the first bin, both male and female participants showed significant group differences between cultural backgrounds, with British children showing longer visit durations. This effect remained significant across all six bins in males, but became non-significant in females. There were also sex differences in British participants, with males showing longer relative visit durations than females in the fourth and sixth bins.

## Discussion

The current study is the first to investigate how cultural background affects how young children observe dynamic faces with either direct or averted gaze, by testing children between the age of 1 and 7 years using different sequences of facial actions. We replicated adults’ finding that British participants fixate more on the mouth, and Japanese participants fixate more on the eyes [15]. The results extends those reported in Kelly et al. [12] by demonstrating that the cultural background starts to affect the face gaze well before the children reach the age of seven. In contrast, we did not replicate the finding in adults that Japanese, but not British, participants flexibly changes eye fixation in response to different gaze directions [15], in young children who participated in the current study. The results suggest that the effect of cultural background on the differential gaze scanning might develop after the age of seven.

The follow-up analyses on the modulatory effect of participants’ sex and age also provide novel and unique insights into the development of culture-specific pattern of face gaze. Firstly, both males and females showed culture-specific pattern of the gaze to the eyes, which contrasts with an adult study in which only males showed this effect. The overall results concerning patterns of mouth gaze replicated adults’ results, with only males showing the culture-specific gaze. A close analysis of second-by-second transition, however, revealed that females also showed similar cultural differences during the first second of stimulus presentation. Secondly, age-related changes manifested differently in male and female children; with the younger male children and the older female children demonstrating the more exaggerated patterns of culture-specific face gaze. This result might suggest the different time-course of the development of face gaze between male and female children. Overall, these results suggest that culture-specific pattern of face gaze is more prevalent, and possibly even more exaggerated, in children than in adults. The current results can also explain why Kelly et al. [10] found that 70% of British born Chinese adults show face fixation pattern similar to Eastern culture, whereas 30% of them show Western pattern of face fixation. If the culture-specific pattern of face gaze develops within the first years of life and diminish over the course of development (or with increasing face-expertise), it is most likely to be affected by the exposure to the culture-specific display in the first years of life from the family and relatives, rather than from peers they encounter later in the life.

Further study will be required to investigate which aspect of cultural background affects the development of differential face gaze between British and Japanese children. A possible explanation might be that such developmental patterns might reflect the social learning of culture-specific displays of facial expression [11,20], that Eastern Asian cultures use dynamic eye movement to display the intensity of (less categorical) emotion, whereas Western European cultures use different sets of facial movements, both in the eyes and in the mouth, to express distinct categories of emotion. Because of these cultural differences, children developing in Eastern Asian cultures would benefit from attending more to the eyes, and children developing Western European cultures would have to monitor both eye and mouth movements to encode others’ emotion. Thus, children developing in each culture might learn the optimal orienting strategies to detect relevant information from early in their life, which is consistent with the current results. It will be beneficial to examine the development of face gaze when young children process the facial expression of each culture, and investigate if they relate to children’s capacity for recognising facial expressions and responding appropriately towards them. Moreover, we only used female faces for our stimuli, as we did not find any effect of the gender of the model on face or gaze scanning [15]. However, it would be beneficial for a further study to confirm if young children change face gaze to male and female faces, and whether it is modulated by the cultural background of the child.

Similarly, we do not yet know why the gaze direction of the actor had no effect on children’s face gaze, in contrast with the adult study [15]. The results might seem surprising, because previous studies demonstrated that young infants can easily recognize different gaze direction [3], and gaze direction modulates the processing of facial identity [21] and facial expression [22]. It is possible to argue that cultural norms on the use of eye contact in face-to-face communication might only be imposed on more 'mature' members of the society. As a result, such a cultural norm won’t start to affect the development of eye gaze until later in the course of development. In addition, we can’t fully rule out the possibility that the difference might be due to the difference in the timing of the presentation of the gaze shift. The current stimuli presented gaze shift 1 s after the presentation of the face, but the previous studies presented different gaze direction from the onset of the stimuli. Note that the design of the current study is different from the situation of gaze following, which involves the presentation of peripheral target stimuli in addition to the face and gaze shift. Further studies will be required to study the developmental trajectory of culturally appropriate pattern of face gaze in response to others’ gaze shift, by testing children older than the current population, as well as adolescents.

Moreover, as in some of the previous studies [9–11], we did not replicate the other-race effect on face scanning (i.e. significant interaction between the ethnicity of the participants and the ethnicity of the stimuli), which contrasts with previous infant research [13,14,23]. It is not clear why some researches find the other race effect and others don’t, a finding that needs to be explored further in future studies.

To summarize, the current study revealed that the cultural background of young children affects how they look at another person’s eyes and mouth, but not the way they modulate eye gaze in response to other people’s eye gaze. The effects are found in both male and female participants and across the age range, but could develop somewhat differently across the lifespan in male and female children. These results highlight the new frontier of the research about how cultural norms can affect the development of social cognition and behaviour, which would provide a great opportunity to study the effect of postnatal environment on human behavioural and cognitive development.
